# Dexamethasone for Inner Ear Therapy: Biocompatibility and Bio-Efficacy of Different Dexamethasone Formulations In Vitro

**DOI:** 10.3390/biom11121896

**Published:** 2021-12-17

**Authors:** Ziwen Gao, Jana Schwieger, Farnaz Matin-Mann, Peter Behrens, Thomas Lenarz, Verena Scheper

**Affiliations:** 1 Lower Saxony Center for Biomedical Engineering, Implant Research and Development (NIFE), Department of Otorhinolaryngology, Head and Neck Surgery, Hanover Medical School, Stadtfelddamm 34, 30625 Hannover, Germany; gao.ziwen@mh-hannover.de (Z.G.); Schwieger.Jana@mh-hannover.de (J.S.); Matin-Mann.Farnaz@mh-hannover.de (F.M.-M.); Lenarz.Thomas@mh-hannover.de (T.L.); 2 Cluster of Excellence “Hearing4all” EXC 1077/2, 30625 Hannover, Germany; peter.behrens@acb.uni-hannover.de; 3 Institute of Inorganic Chemistry, Leibniz University Hannover, 30167 Hannover, Germany

**Keywords:** cochlear implant, dexamethasone, LPS, TNF-α, fibrosis, anti-inflammatory, drug delivery, biocompatibility, MTT test

## Abstract

Dexamethasone is widely used in preclinical studies and clinical trials to treat inner ear disorders. The results of those studies vary widely, maybe due to the different dexamethasone formulations used. Laboratory (lab) and medical grade (med) dexamethasone (DEX, C_22_H_29_FO_5_) and dexamethasone dihydrogen phosphate-disodium (DPS, C_22_H_28_FNa_2_O_8_P) were investigated for biocompatibility and bio-efficacy in vitro. The biocompatibility of each dexamethasone formulation in concentrations from 0.03 to 10,000 µM was evaluated using an MTT assay. The concentrations resulting in the highest cell viability were selected to perform a bio-efficiency test using a TNFα-reduction assay. All dexamethasone formulations up to 900 µM are biocompatible in vitro. DPS-lab becomes toxic at 1000 µM and DPS-med at 2000 µM, while DEX-lab and DEX-med become toxic at 4000 µM. Bio-efficacy was evaluated for DEX-lab and DPS-med at 300 µM, for DEX-med at 60 µM, and DPS-lab at 150 µM, resulting in significantly reduced expression of TNFα, with DPS-lab having the highest effect. Different dexamethasone formulations need to be applied in different concentration ranges to be biocompatible. The concentration to be applied in future studies should carefully be chosen based on the respective dexamethasone form, application route and duration to ensure biocompatibility and bio-efficacy.

## 1. Introduction

More than 5% of the world’s population is living with a hearing disability [1,2]. To date, the exact mechanisms of sensorineural hearing loss (HL) are still not completely understood [3] but in preclinical and clinical otology fields, in vitro and in vivo studies and clinical trials are often conducted to seek pharmacotherapies of inner ear disorders [4,5,6]. Among these, inflammation-suppressing substances, such as glucocorticoids, and especially dexamethasone, have been widely tested as a potential therapy to treat inner ear pathologies including sudden sensorineural hearing loss (SSNHL) [7,8,9,10], Menière’s disease [11,12,13,14], and acute tinnitus [15]. Additionally, dexamethasone is intensively investigated for its benefit in cochlear implant (CI) patients [16]. Today, for patients with a severe-to-profound SNHL or deafness, independently of age, CIs are the most effective treatment. Moreover, patients with remaining residual hearing in low frequencies are treated by CI [17]. The CI electrode array is implanted into the cochlea of the inner ear, stimulating the primary auditory neurons and therefore eliciting a hearing sensation. The benefit in speech perception and hearing of noises that patients have with a CI is tremendous, but in some cases, insertion trauma and foreign body reaction lead to an inflammation. This inflammatory reaction per se may negatively affect the intention of preserving the residual hearing. Inflammatory mediators, a subsequent fibrosis around the electrode or even newly grown bone may dampen the cochlear mechanics and therefore reduce the residual hearing [18,19,20]. The main compound used in research regarding the optimization of fibrosis reduction and residual hearing preservation in CI is dexamethasone. Several studies have concluded that dexamethasone has a suppressing effect on cochlear tissue inflammation and improves hearing preservation after CI surgery. Dexamethasone inhibits a tumor necrosis factor-α (TNFα) initiated inflammatory response of spiral ligament fibrocytes in vitro [21] and reduces electrode impedances and fibrose tissue growth around the electrode array in an animal model of electrode insertion trauma [22]. Additionally, dexamethasone protects hair cells of Organ of Corti explants that were challenged with an ototoxic level of TNF-α [23] and protects the hearing ability in guinea pig models of cochlear implantation [24,25,26,27] and in non-human primates [28]. In contrast, others report no effect or negative effects of dexamethasone on inner ear tissue and function. Some studies report that dexamethasone has no effect on fibrosis in cochlear implanted guinea pigs [29,30,31]. Toxic effects on cultured hair cells have been reported [32] and dexamethasone-treated guinea pigs either had an increased hearing loss at 8, 16, and 32 kHz compared to control animals [22] or the hearing-preserving effect lasted for a limited time only [3]. Therefore, the biological effects of dexamethasone for inner ear therapy vary widely. This may be due to the applied concentrations, the application route and duration, the extent of intracochlear trauma and the time point of readout after therapy. Additionally, the nomenclature for different dexamethasone formulations in publications on inner ear therapy is often not correct [33]. We hypothesize that the variation of dexamethasone therapy of the inner ear is, amongst other reasons, based on the different dexamethasone formulations used. A systematic investigation of favorable formulations to be used for inner ear drug delivery and therapy is the first step towards evaluating the biocompatibility and bio-efficacy of different dexamethasone formulations. The aim of the current study is to examine whether different formulations of dexamethasone show different levels of in vitro cytotoxicity and also differences in bio-efficacy. To our knowledge, this is the first report on the concentration-dependent biocompatibility and bio-efficacy of different dexamethasone formulations. The findings may help to determine the right dexamethasone formulation and concentration to be applied to the cochlea to treat SSNHL, Menière´s disease, tinnitus or cochlear implant-related pathologies.

## 2. Materials and Methods

### 2.1. Dexamethasone Formulations

The dexamethasone formulations tested in this study were chosen based on previous studies reporting details for inner ear therapy. Only a few publications exactly name the formulation, molecular weight and provider [33]. Two different dexamethasone molecules are used in published studies: pure dexamethasone (DEX) and dexamethasone dihydrogen phosphate disodium (DPS). For each formulation, one product of laboratory standard (DEX-lab; DPS-lab) and one of pharmaceutical grade (DEX-med; DPS-med) were chosen and included in this study. Table 1 lists the different dexamethasone formulations and corresponding abbreviations used, the molecule formula, the molecular weight, the CAS-No. (Chemical Abstracts Service), the manufacturer, related article number and comments.

### 2.2. Cell Lines and Culture Conditions

#### 2.2.1. Biocompatibility Test

**Figure 1:**
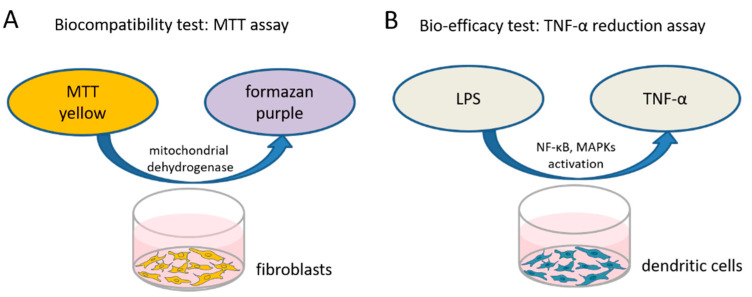
To test the biocompatibility of the various dexamethasone formulations and concentrations an MTT assay was performed. (**A**) Living, metabolically active fibroblasts reduce the MTT to formazan, changing the color of the medium from yellow to purple. This color change is quantified by measuring the optical density using a microplate reader. The capability of the tested dexamethasone formulations to affect inflammatory reactions is exemplarily tested on dendritic cells which produce a high amount of TNFα when stressed with lipopolysaccharides (LPS). (**B**) If dexamethasone has an anti-inflammatory effect it should reduce the TNFα-production, which is measured using an ELISA.

To test the biocompatibility of the different dexamethasone formulations in various concentrations a 3-(4,5-dimethylthiazol-2-yl)-2,5-diphenyltetrazolium bromide (MTT)-assay (PanReac AppliChem, Darmstadt, Germany) was performed to measure the cellular metabolic activity. The reagent MTT binds to the mitochondria of living cells and is enzymatically reduced to a blue/purple product (Figure 1A). The detected color intensity is related to the number of viable cells, and is thus related directly to their proliferation in vitro. Mouse NIH/3T3 fibroblasts (German Collection of Microorganisms and Cell Cultures GmbH, Braunschweig, Germany) (passage 3 to 10) from our in-house stock were standardly cultured in Dulbecco’s modified Eagle’s medium (DMEM, Bio and Sell GmbH, Feucht, Germany) supplemented with 10% fetal calf serum (FCS, Bio and Sell GmbH, Feucht, Germany), penicillin, and streptomycin (100 units/mL each) in a humidified atmosphere of 5% CO_2_/95% air at 37 °C. To perform the MTT assay, the fibroblasts were seeded in 96-well plates at a concentration of 1.5 × 10^4^ cells/mL, supplied with 100 µL fresh culture medium and incubated. After 24 h, the culture medium was changed and various concentrations of the different dexamethasone formulations (DEX-med, DEX-lab, DPS-med, DPS-lab) were included.

An orientation study was performed using low concentrations from 3 to 600 µM. The results of those tests (*N* = 3, *n* = 3) were taken to determine the concentration to be used in the bio-efficacy test (see below). Additionally, this orientation study showed that a wider range of concentrations have to be tested for cell toxicity. In subsequent experiments, concentrations from 0.03 to 10,000 µM were tested in the MTT test (Table 2).

Cells treated with 0.1% DMSO served as positive control (PC) for a toxic effect on the cells while the negative control (NC) cells were cultured in pure complemented medium for normal cell proliferation. The PC and NC conditions run in parallel with each single experiment to validate that the experiment was performed successfully. All experiments were performed in triplicate and repeated three times. After 24 h, the medium was removed, replaced by 50 µL 0.5 mg/mL MTT reagent and incubated for two hours at humidified atmosphere of 5% CO_2_/95% air at 37 °C. Subsequently, the MTT reagent medium was removed and replaced by 100 μL MTT solution (isopropanol) per well and incubated for five minutes on a rotary shaker at room temperature to dissolve the formazan produced by MTT reduction. In order to quantify the cell viability (CV), absorbance measurements were performed at a wavelength of 570 nm using a MicroPlate Reader (Gen5 2.06.Ink, BioTek Synergy™ H1HyBrid Reader, Santa Clara, CA, USA) to detect the optical density (OD). The mean measured value of three wells was set as blank dataset (ODb; MTT reagent solved in culture medium in cell-free wells) and subtracted from the detected absorbance values of the dexamethasone treated cells (OD_DEX_), obtaining the relative cell viability (CV%) with respect to the negative control (ODnc). To finally calculate the CV% for the wells of tested samples (ODt), dexamethasone- and DMSO-treated cells, the blank-corrected ODb was divided by the blank-corrected ODnc of the negative control and multiplied by 100. The following equation was applied:

$$\mathrm{CV}\%=\frac{\mathrm{ODt}-\mathrm{ODb}}{\mathrm{ODnc}-\mathrm{ODb}}\times 100$$

Cytotoxicity was assessed based on the calculated CV, using a classification system adapted from [34] and according to annex C of ISO 10993-5 for the biocompatibility testing of medical devices, which states that a treatment resulting in a CV% under 70% of the NC has cytotoxic potential [35]. The MTT results were analyzed for the concentration with the highest CV and thus the best biocompatibility for each formulation, which was subsequently used for the bio-efficacy testing.

#### 2.2.2. Bio-Efficacy

TNF-α is a multifunctional signaling substance of the immune system that is involved in local and systemic inflammation. Intracochlear inflammation and hair cell damage are associated with TNF-α expression [36]. The TNF-α reduction assay is commonly used to investigate anti-inflammatory potential of therapy strategies [37]. In this assay, cells are stressed by adding LPS (Sigma-Aldrich, St. Louis, MO, USA) and produce, in response to the stress, TNF-α. We tested the bio-efficacy of the previously verified biocompatible concentrations of each dexamethasone formulation on TNF-α production after LPS-induced stress on dendritic cells as model cell line (Figure 1B). The level of TNF-α expression was analyzed using an enzyme-linked immunosorbent assay (ELISA).

**Figure 2:**
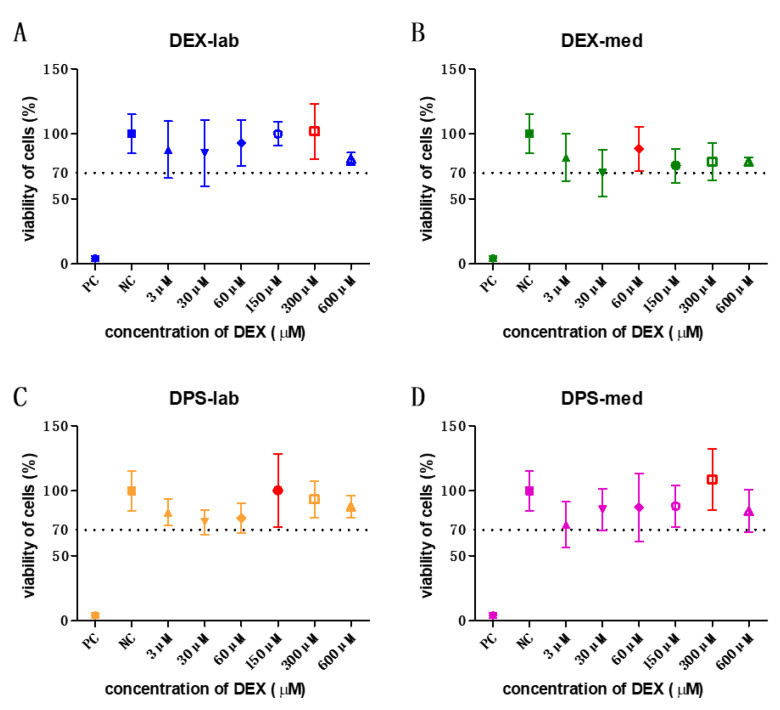
All tested concentrations of the orientation study were biocompatible. The results of these experiments were taken to decide the concentration to be used in the bio-efficacy tests. The highest mean viability of cells is labelled in red in each graph. The dotted line indicates the 70% viability rate.

Cells of the DC2.4 mouse dendritic cell line (DCs) (Sigma-Aldrich, St. Louis, MO, USA, LOT:3093896) were maintained in RPMI 1640 medium (Sigma-Aldrich, St. Louis, MO, USA) supplemented with non-essential amino acids (1 mmol/L, Sigma-Aldrich, St Louis, MO, USA) and 10% FCS (Bio & Sell GmbH, Feucht, Germany). The cells were cultured in a humidified atmosphere of 5% CO_2_/95% air at 37 °C. All experiments were performed using cells of passage 3 to 10. DCs were seeded at a density of 1.5 × 10^5^ cells/mL in 48-well plates (Eppendorf, Hamburg, Germany). Each well contained 200 µL of the medium and the cells were cultivated for 24 h in an incubator. Thereafter, the wells were divided into negative control (NC), positive control (PC) and dexamethasone groups. Cells of the PC-wells were stimulated with 100 µL LPS included in the medium (0.5 µg/mL), while cells with plain DC-medium served as the NC. The PC and NC conditions were run in parallel with each single experiment to validate that each experiment was performed successfully. Treatments with 300 µM DEX-lab, 60 µM DEX-med, 150 µM DPS-lab, and 300 µM DPS-med (see Figure 2), respectively, combined with LPS-stress were applied to the experimental groups. All groups were incubating for additional 24 h. Treatments were performed in duplicate per plate (*n* = 2) for every independent experiment (*N* = 3). After 24 h, the supernatants of each well were collected and stored at −20 °C for subsequent ELISA analysis.

#### 2.2.3. TNF-α Detection

The collected supernatants were analyzed for the TNF-α protein concentration using ELISA kits (Boster Biological Technology, CA, USA) according to the manufacturer’s instructions. Each supernatant was applied to the ELISA plate in dilution and as a replicate. OD absorbance was recorded at 450 nm using a MicroPlate Reader (Gen5 2.06.Ink, BioTek Synergy™ H1HyBrid Reader, Santa Clara, CA, USA). The TNF-α concentration in the supernatants was calculated after blank-subtraction by comparison with a standard curve generated using the manufacturer-provided standard and Gen5™ 2.06.Ink software.

#### 2.2.4. Statistical Analysis

The effects of the dexamethasone formulations and concentrations on the viability of cells and the level of TNF-α production after LPS-stress were analyzed using GraphPad Prism^®^ version 8.4.3. Data were checked for normal distribution using the D’Agostino and Pearson normality test. Differences in CV between NC and dexamethasone treatment were analyzed using one-way ANOVA followed by Bonferroni’s multiple comparison test. An unpaired t-test was used to determine the effects on TNF-α production between the PC and a given dexamethasone formulation. The data are reported as mean ± standard deviation (SD). Statistical significance was considered at *p* values less than 0.05. 

## 3. Results

### 3.1. Cell Viability for Biocompatibility

The orientation study revealed that the tested concentrations of 3, 30, 60, 150, 300, and 600 µM were biocompatible (Figure 2) according to annex C of ISO 10993-5 for biocompatibility testing. Based on these results, the dexamethasone concentrations with the highest mean value per formulation were chosen for the bio-efficacy tests (Figure 2, red bars). For DEX-lab, a concentration of 300 µM (mean: 101.9% CV), DEX-med 60 µM (mean: 88.53%), DPS-lab 150 µM (mean: 100.4%), and DPS-med 300 µM (means: 108.7%) were tested. 

**Figure 3:**
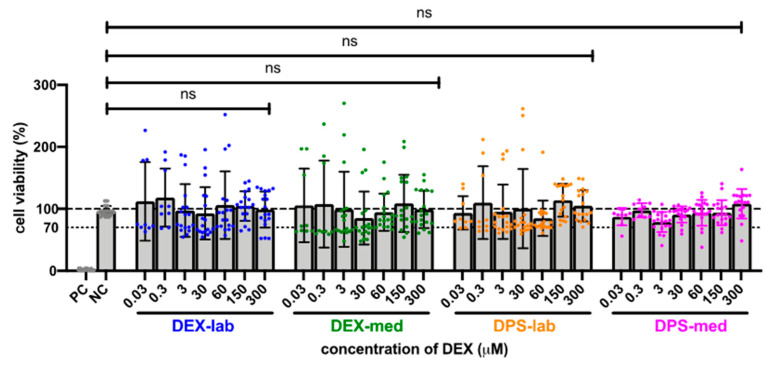
Comparison of cell viability (CV in %) of fibroblasts treated with low concentrations (0.03–300 µM) of four different dexamethasone formulations. The CVs of the tested low concentrations within one tested dexamethasone formulation did not differ. No significant differences were detected between the NC and any low concentration DEX-treatment. The dashed line indicates 100% CV. The dotted line at 70% CV marks the toxicity level, based on the ISO guideline for biocompatibility testing of medical devices. The tested dexamethasone formulations and concentrations all resulted in cell vitality above the toxicity level. Data are given as mean ± SD in bar charts with single experimental results included as dots (*N* = 3, *n* = 3); ns = not significant.

Since the results of the orientation study showed that there was no toxic effect at 600 µM, the tested dexamethasone concentrations were increased to 10,000 µM to detect a toxicity limit. For better clarity, the results of the expanded MTT test are presented in two concentration groups for the various dexamethasone formulations: low concentrations ranging from 0.03 to 300 µM, and high concentrations ranging from 600 to 10,000 µM. The NC was set at the normal cell proliferation with CV% of 96.79 ± 7.718% and the included PC with DMSO treatment of the fibroblasts revealed a clear cytotoxic effect in the MTT test with a massive decreased CV% of 1.95 ± 1.30% in the performed experiments. The mean of all the low concentrations tested from 0.3 to 300 resulted in an average CV for DEX-lab of 108.9 ± 51.59%, for DEX-med of 113.3 ± 55.48% and for DPS-lab and DPS-med of 110.0 ± 49.67% and 96.15 ± 17.52%, respectively (Figure 2). A comparison within the tested low concentrations of each dexamethasone formulation showed no statistically significant difference (Figure 3; Table 3). Additionally, there was no significant difference detectable between the CVs of NC and the dexamethasone treated cells. None of the low concentrations of the dexamethasone formulations reduced the CV below 70%. According to annex C of ISO 10993-5 for the biocompatibility testing of medical devices, this suggests that all the named low concentrations tested were biocompatible. 

**Figure 4:**
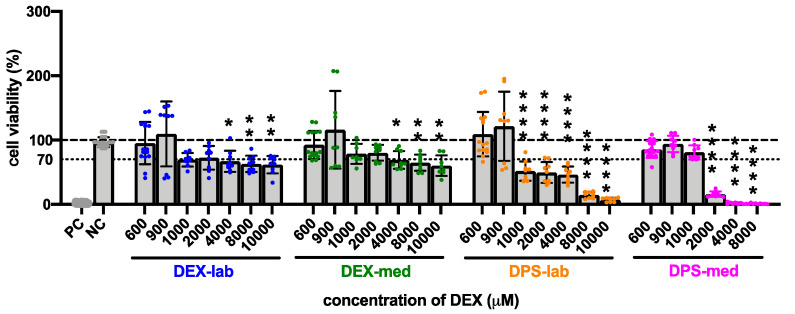
Influence of higher (600 to 10,000 µM) concentrations of dexamethasone formulations on cell viability (CV) detected by MTT assay. The dashed line indicates 100% CV. A reduction of the CV below the 70% level (dotted line) indicates a cytotoxic effect. At a concentration of 4000 µM and higher, the CV was significantly reduced in all treatment groups when compared to the NC. For DPS-lab this was already the case at a concentration of 1000 µM and for DPS-med this was already the case at 2000 µM. Data are given as mean ± SD in bar charts, with single experimental results included as dots (*N* = 3, *n* = 3). Significant differences to the NC are indicated by * (*p* < 0.05), ** (*p* < 0.01) and **** (*p* < 0.0001).

The results of the statistical analysis of the CV of fibroblasts treated with higher dexamethasone concentrations (600 to 10,000 µM) compared to the NC are summarized in Table 3 and Figure 4. Initially, none of the dexamethasone formulations in concentrations of 600 and 900 µM has an effect on cell vitality. At higher concentrations, the various formulations became toxic. Dexamethasone dihydrogen phosphate disodium is already toxic at 1000 µM (DPS-lab, 51.73 ± 15.08%) and 2000 µM (DPS-med, 15.90 ± 4.36%). For pure dexamethasone, cell viability fell below the 70% CV limit at 4000 µM (DEX-lab and DEX-med, 67.12 ± 16.45% and 68.97 ± 13.71%). Overall, in the higher concentrations the toxicity is higher for DPS, with an average CV for DPS-lab and DPS-med of 7.26 ± 3.06% and 1.13 ± 0.79%, respectively, as compared for DEX, which had a CV for DEX-lab and DEX-med of 61.82 ± 13.55% and 60.16 ± 15.91%, respectively.

### 3.2. Bio-Efficiency Evaluation by TNF-α Detection

**Figure 5:**
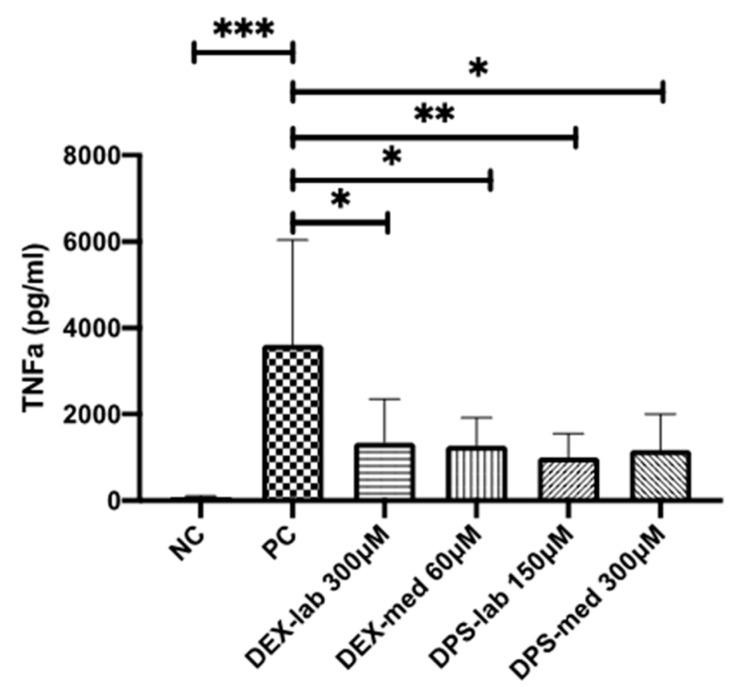
TNF-α amounts measured by ELISA in the supernatants of dendritic cells (DCs). TNF-α production is induced by addition of 0.5 µg/mL LPS to the culture medium. This results in a high release of TNF-α in the PC when compared with the basic TNF-α level of unstressed cells in the NC. All tested dexamethasone formulations reduced the TNF-α amount in culture. Data are given as mean ± SD and detected significances are marked with * (*p* < 0.05), ** (*p* < 0.01), and *** (*p* < 0.001).

The potential of the different dexamethasone formulations to reduce an inflammatory response was analyzed by means of TNF-α production, measured by ELISA-detection after LPS-stress of the dendritic cells (DCs) (Figure 5). The dexamethasone concentrations with the best biocompatibility were added to the DCs: 300 µM DEX-lab, 60 µM DEX-med, 150 µM DPS-lab, and 300 µM DPS-med. The DCs cultured with pure medium (NC) produced a basic TNF-α level of 91.35 ± 18.73 pg/mL (mean ± SD). Adding 0.5 µg/mL LPS to induce cell stress (PC) increased the TNF-α amount in the supernatant to 3611 ± 2425 pg/mL and thus significantly compared to the NC (*p* = 0.001). Compared to the PC, all tested dexamethasone formulations reduced the measured TNF-α amount in the culture supernatants significantly (*p* = 0.0451 for DEX-lab, DEX-med and DPS-med; *p* = 0.0016 for DPS-lab). The TNF-α level was 1335 ± 1013 pg/mL for DEX-lab, 1272 ± 646.3 pg/mL for DEX-med, 1000 ± 552.4 pg/mL for DPS-lab, and 1169 ± 832.4 for DPS-med.

## 4. Discussion

Glucocorticoids are considered to have great therapeutic potential for inner ear diseases. This class of steroidal hormones includes, prednisone, triamcinolone, cortisone, and dexamethasone, and dexamethasone is the most popular for use in the field of inner ear therapy. Although many studies highlight the therapeutic effect of dexamethasone in the inner ear (e.g., [24,26,27]), factors such as the concentration to be applied or the delivery route affect its biological effects. Next to this the chemical compound composition may be one reason for the varying in vitro, in vivo and clinical study results.

To our knowledge, no published study has focused on investigating the biocompatibility of different types of dexamethasone in a direct comparison of a dilution series. This lack of information is intended to be remedied by the data presented here. We tested the biocompatibility and bio-efficacy of the two dexamethasone formulations, dexamethasone and dexamethasone dihydrogen phosphate disodium, which have previously been used in hearing research (Table 4). Two products were selected for both formulations in this study: one that was laboratory grade and one that can potentially be transferred as a therapeutic to the clinic as it is pharmaceutical grade. Concentrations from 0.03 µM to 10,000 µM were selected for biocompatibility testing.

All types of dexamethasone were biocompatible in lower concentrations (0.03–300 µM; Figure 3). However, with increasing concentrations of dexamethasone, the viability of the cells in each group decreased, albeit to a differing extent. DEX-lab and DEX-med were safe up to 2000 µM (784 µg/mL; 0.0784 mg/mL), while DPS treatment was only safe up to 900 µM (464 µg/mL; 0.464 mg/mL; DPS-lab) and 1000 µM (516 µg/mL; 0.516 mg/mL; DPS-med). To shed light on our results with regard to the current state of knowledge we randomly reviewed literature presenting results for the usage of dexamethasone in cochlear pharmacotherapy (Table 4).

The concentrations listed in Table 4 cover the full range of concentrations we tested in our biocompatibility MTT assay. Studies using dexamethasone concentrations below the concentrations we detected to be toxic in vitro (i.e., <0.5 mg/mL for DPS and <1.6 mg/mL for DEX) did not report the biological effects of low concentrations on hearing ability (Conolly et al. 2011: 0.002 mg/mL DEX-lab i.v. prior to CI surgery; Taketa et al., 2021: 0.002 mg/mL DEX-lab; Lyu et al., 2018: 0.01 mg/mL DPS lab), but biocompatibility and a protective effect on SGN if applied in parallel with cochlear implant based electrical stimulation was reported (Scheper et al., 2017: 0.1 µg/mL pump based [30]). Concentrations which were cell compatible in our experiments (below 600 µM) were already classified as toxic in previous reports (0.00117 mg/mL = 1.1177 µg/mL = 3 µM DEX-med in vitro [32]). In contrast, concentrations which significantly reduced the CV in our in vitro tests were already shown to have a beneficial effect on residual hearing in animal models, not being toxic in vivo. For example, 5 mg/mL (DPS-lab, in vivo, i.c, i.t., [43]) is much higher than the concentration detected to be toxic in our study (DPS-lab: 900 µM = 0.464 mg/mL). The highest concentration was used by Alexander et al. (2015), where intratympanically injected 24 mg/mL DPS-lab resulted in pure tone hearing threshold recovery after SSNHL and was massively above the concentrations having a toxic effect in our study for the different dexamethasone formulations. 

Even though we aimed to compare the listed concentrations (Table 4) to our results, we admit that such a comparison is hardly possible since the reported dexamethasone amounts were mostly not the relevant concentrations in the inner ear but the drug load of a matrix or a concentration applied systemically. Therefore, the dexamethasone concentrations achieved in the scala tympani are mostly unknown. 

The individual parameters of the different treatment protocols, like intravenous or intraperitoneal injection, systemic therapy, or drug release of a matrix such as the CI electrode array make it difficult to directly compare the outcomes of the studies, especially with respect to the actual dose of DEX delivered.

The best CV rate was detected for DEX-lab and DPS-med at 300 µM, for DEX-med at 60 µM, and for DPS-lab at 150 µM. Those optimal concentrations were compared regarding their anti-inflammatory effect. No differences were observed between the formulations but there was a slight tendency for DPS-lab to be more effective in reducing TNF-α production than the other formulations. This suggests that different dexamethasone formulations may achieve similar anti-inflammation effects in the inner ear. A future study should address this topic by investigating the anti-inflammatory potential of different dexamethasone formulations in a set up involving accelerating concentrations to be able to suggest the most promising dexamethasone formulation and respective concentration to reduce anti-inflammatory reactions in general, and in the inner ear in specific.

The choice of dexamethasone to be used and the concentration to be applied for inner ear pharmacotherapy was guided by different questions. What is the biological effect one is aiming for: hearing preservation, fibrosis reduction, SGN protection or a general reduction of inflammatory reactions? All dexamethasone formulations tested in this study have an anti-inflammatory effect, as has been described in literature in general for dexamethasone. Which concentrations have to be reached locally in the inner ear to receive the aimed effects was not previously known. 

What will the delivery matrix be? Drug delivery systems are needed for the sustained treatment of inner ear diseases. Whereas a hydrogel-based delivery would favor DPS-lab because of its hydrophilicity [27,30] DEX-med is used for delivery through hydrophobic materials such as silicone [22,26] or PLGA [45].

Where is the matrix placed to release the DEX? If it is inserted into the inner ear, a direct release into the perilymph is possible, while with intratympanic application the drug has to pass the round window membrane, which affects the concentration to be reached in the inner ear [46].

Which concentration should be chosen? This is a trickier question as it is not known which concentration is needed in vivo to achieve a biological effect. As listed in Table 4, the biologically effective concentrations ranged from 0.00117 mg/mL [32] to 24 mg/mL [42]. Based on our results and in view of Table 4, we concluded that the concentrations need to be chosen with respect to the DEX formulation used, since there are massive differences in cytotoxicity in vitro (900 µM (DPS-lab) versus 2000 µM (DEX-med and DEX-lab)). There is a large variability between concentrations being toxic in vivo and those having a beneficial effect. This could be attributed to the variations of the different treatment protocols. Individual parameters regarding the route of administration (intracochlear, intratympanic, intraperitoneal or intravenous), single shot injection or permanent infusion, delivery matrix, release kinetics, species and the trauma model used affect the DEX effect. With the data available until now, it was not possible to recommend one concentration for a specific DEX formulation to be used in clinical trials, since the in vitro and in vivo studies were too heterogeneous. Since animal trials need to be ethically justifiable, are time-consuming and costly, in silico trials are needed to harmonize the available data and to generate data sets which allow a decision for the most promising combination of DEX formulation and concentration, delivery route and therapy duration to induce relevant biological effects on our patients. 

## 5. Conclusions

Different dexamethasone formulations need to be applied in different concentration ranges to be biocompatible. All forms tested in the respective biocompatible concentrations reduced TNFα-production, indicating that they may have anti-inflammatory capacity in vivo. Therefore, we recommend choosing the concentration to be applied in future studies carefully, based on the respective dexamethasone form, to ensure biocompatibility and bio-efficacy. Future studies should elucidate which effects the various dexamethasone formulations, concentrations, administration routes and durations of treatment have in the inner ear. Improvements of the dexamethasone effects and reliable outcomes in inner ear therapy will only be achieved if structured experiments and in silico trials with a comparison of the treatment variations are conducted and transferred into clinical studies.

## Figures and Tables

**Table 1:** Characteristics of the different dexamethasone formulations used in this study.

Abbreviation	Formula	Molecular Weight(g/mol)	CAS-No.	Manufacturer, Article-No.	Comment
DEX-med	C_22_H_29_FO_5_	392.46	50-02-2	Caesar & Loretz GmbH, Hilden, Germany; 2211	Recipe substance for pharmaceutical formulations or active pharmaceutical ingredients; powder
DEX-lab	C_22_H_29_FO_5_	392.46	50-02-2	Sigma-Aldrich, St. Louis, MO, USA; PHR1526	Laboratory chemical; powder
DPS-med	C_22_H_28_FNa_2_O_8_P	516.4	50-02-2	MerckSerono, Darmstadt, Germany; 7880135315	Fortecortin^®^ Inject 4 mg; approved drug, solution
DPS-lab	C_22_H_28_FNa_2_O_8_P	516.4	2392-39-4	Sigma-Aldrich, St. Louis, MO, USA; D0720000	Laboratory chemical, manufactural substances; powder

**Table 2:** List of the different dexamethasone formulations tested in the indicated concentrations, reported in molecular weight, and respective concentration, reported as mg/mL and µg/mL.

DEX Formulation				
Concentration	DEX-Med, DEX-Lab; 392.46 g/mol		DPS-Med, DPS-Lab; 516.4 g/mol	
µM	mg/mL	µg/mL	mg/mL	µg/mL
0.03	1.18 × 10^−5^	0.0118	1.55 × 10^−5^	0.0155
0.3	1.18 × 10^−4^	0.1177	1.55 × 10^−4^	0.1549
3	1.18 × 10^−3^	1.177	1.55 × 10^−3^	1.5492
30	1.18 × 10^−2^	11.77	1.55 × 10^−2^	1.5492
60	2.36 × 10^−2^	23.55	3.10 × 10^−2^	30.984
150	5.89 × 10^−2^	23.55	7.75 × 10^−2^	77.46
300	0.118	117.74	0.155	154.92
600	0.235	235.48	0.310	154.92
900	0.353	353.21	0.465	464.76
1000	0.392	392.46	0.516	516.4
2000	0.784	784.92	1.03	1032.8
4000	1.60	1569.84	2.07	2065.6
8000	3.14	3139.68	4.13	4131.2
10,000	3.92	3924.6	5.16 *	5164 *

*: In the DPS-med group, 10,000 µM was not tested because the solubility limit was exceeded at 4 mg/mL.

**Table 3:** *p* value results of the statistical analysis of the CV data compared to negative control.

	Concentration (µM)							
	0.03–300	600	900	1000	2000	4000	8000	10,000
DEX-lab	n.s.	0.9998	0.9546	0.0562	0.0990	0.0140	0.0024	0.0013
DEX-med	n.s.	0.9994	0.4153	0.6045	0.6919	0.0293	0.0053	0.0014
DPS-lab	n.s.	0.8198	0.0926	<0.0001	<0.0001	<0.0001	<0.0001	<0.0001
DPS-med	n.s.	0.9161	0.9997	0.7436	<0.0001	<0.0001	<0.0001	-

n.s. = not significant (*p* > 0.05) compare to the negative control; grey: highlighted statistically relevant differences compared to the negative control; DPS-med was not tested in 10,000 µM because the stock solution had a lower concentration.

**Table 4:** List of studies using dexamethasone for inner ear therapy.

Dexamethasone Formulation, Molecular Weight	Reference	Study Type	Delivery Method	Concentration (mg/mL) *	Remarks
DEX-lab, 392.46 g/mol	Connolly et al., 2011 [24]	In vivo	i.v. prior to CI	0.0002; 0.002	Lower dose failed to maintain ABR thresholds. High-dose treatment resulted in a reduction of ABR threshold shift.
	Kuthubutheen et al., 2014 [6]	In vivo	i.p.	0.002	Spiral ganglion neuron (SGN) density was increased compared to traumatized controls.
	Jia et al., 2016 [32]	In vitro; in vivo	explants; pump based delivery (1 µL/h, 7 days);	0.00117; 0.0117; 0.117; 0.117	In vitro: 0.00117 and 0.0117 mg/mL start to have toxic effects on outer hair cells, 0.117 mg/mL is toxic for inner and outer hair cells; in vivo: 0.117 mg/mL is toxic for SGN but improves ABR thresholds at selected frequencies.
	Takeda et al., 2021 [14]	In vivo	i.p.	0.002	No effect.
DEX-med, 392.46 g/mol	Serrano Cardona et al., 2013 [38]	Clinical	DEX in PLGA polymer	0.7	Mean hearing threshold improved.
	Bas et al., 2016 [26]	In vivo	DEX in CI silicone; 0.1% = 13 ng/day, 1.0% = 60 ng/day and 10% = 161 ng/day	1; 10; 100	10% and 1.0% protected against electrode insertion-induced HC loss, but increased ABR and CAP thresholds and impedance, fibrosis and loss of cochlear nerve elements.
	Wilk et al., 2016 [22]	In vivo	DEX in CI silicone (16 ng/day and 49 ng/day)	10; 100	Reduced impedances and fibrous tissue growth; increased hearing thresholds.
	Scheper et al., 2017 [30]	In vivo	DEX in CI silicone (16 ng/day and 49 ng/day; i.e., 0.66 ng/h and 2.04 ng/h)	10; 100	Normal SGN number and increased soma diameter.
	Ahmadi et al., 2019 [39]	In vivo	6% DEX loaded hydrogel and DEX containing CI	60	Auditory nerve fiber protection.
DPS-lab, 516.40 g/mol	James et al., 2008 [40]	In vivo	i.t.; 5 µL of 2%	20	Residual hearing preservation.
	Souter et al., 2009 [41]	In vivo	i.t., 20% in sponge	200	Hearing protection at lower concentrations.
	Hütten et al., 2014 [27]	In vitro, In vivo	StarPEG-hydrogel filled reservoir, (50 µg DEX/µL hydrogel, 0.35 μg DEX/h)	50; 50	Hearing protection, reduced fibrosis.
	Alexander et al., 2015 [42]	Clinical	DEX i.t., four injections in two weeks	10; 24	Recovery of hearing threshold after SSNHL.
	Scheper et al., 2017 [30]	In vivo	StarPEG-hydrogel filled reservoir, (50 µg DPS-lab/µL hydrogel, 0.35 μg DEX/h)	50	Biocompatible regarding SGN number and soma diameter.
	Lyu et al., 2018 [43]	In vivo	i.c., i.t. and i.p.	5; 5; 0.01	5 but not 0.01 mg/mL preserved hearing in cochlear implanted animals.
	Ahmadi et al., 2018 [39]	Clinical	Temporarily implanted catheter (4 mg/mL/day)	4	No effect.
DPS-med, 516.40 g/mol	Coimbra et al., 2007 [44]	In vivo	i.p. every 8 h	0.0007	Not effective in preventing neuron loss in pneumococcal meningitis-induced hearing loss.
	Berjis et al., 2016 [9]	Clinical	i.t. (4 mg/mL/day)	4	Hearing improvement.
	Scheper et al., 2017 [30]	In vivo	osmotic pump (25 pg/h)	0.0001	Biocompatible regarding SGN number, decreased soma diameter; with electrical stimulation: increased SGN number.

*: most references do not state mg/mL. This information was calculated using the relevant information of the respective publication; i.v.: intravenous; i.p.: intraperitoneal; i.t.: intratympanic; i.c.: intracochlear.

## Data Availability

All data are available upon request.

## Abbreviations

CI	Cochlear implant
DEX-lab	Dexamethasone—laboratory grade
DEX-med	Dexamethasone—medical grade
DPS-med	Dexamethasone dihydrogen phosphate-disodium—medical grade
DPS-lab	Dexamethasone sodium phosphate—laboratory grade
DMEM	Dulbecco’s modified Eagle’s medium
ELISA	Enzyme-Linked Immunosorbent Assay
FCS	Fetal calf serum
FBs	NIH/3T3 mouse fibroblasts
h	hour
HL	Hearing loss
i.c.	Intracochlear
i.p.	Intraperitoneal
i.t.	Intratympanic
i.v.	Intravenous
LPS	Lipopolysaccharides
MTT	3-(4,5-dimethylthiazol-2-yl)-2,5-diphenyltetrazolium bromide
NC	Negative control
OD	Optical density
PC	Positive control
RT	Room temperature
SSNHL	Sudden sensorineural hearing loss
SGN	Spiral ganglion neurons
SD	Standard deviation
TNF-α	Tumor necrosis factor alpha
